# The NOX Family of Proteins Is Also Present in Bacteria

**DOI:** 10.1128/mBio.01487-17

**Published:** 2017-11-07

**Authors:** Christine Hajjar, Mickaël V. Cherrier, Gaëtan Dias Mirandela, Isabelle Petit-Hartlein, Marie José Stasia, Juan C. Fontecilla-Camps, Franck Fieschi, Jérôme Dupuy

**Affiliations:** a Univ. Grenoble Alpes, CEA, CNRS, IBS, 38000 Grenoble, France; b Univ. Grenoble Alpes, CNRS, TIMC-IMAG, 38000 Grenoble, France; c CDiRec, Pôle Biologie, CHU of Grenoble Alpes, Grenoble, France; Duke University School of Medicine

**Keywords:** *Streptococcus pneumoniae*, biochemistry, electron transport, flavoenzymes, membrane proteins, metalloenzymes, oxidative stress, phylogenetic analysis

## Abstract

Transmembrane NADPH oxidase (NOX) enzymes have been so far only characterized in eukaryotes. In most of these organisms, they reduce molecular oxygen to superoxide and, depending on the presence of additional domains, are called NOX or dual oxidases (DUOX). Reactive oxygen species (ROS), including superoxide, have been traditionally considered accidental toxic by-products of aerobic metabolism. However, during the last decade it has become evident that both O_2_^•−^ and H_2_O_2_ are key players in complex signaling networks and defense. A well-studied example is the production of O_2_^•−^ during the bactericidal respiratory burst of phagocytes; this production is catalyzed by NOX2. Here, we devised and applied a novel algorithm to search for additional *NOX* genes in genomic databases. This procedure allowed us to discover approximately 23% new sequences from bacteria (in relation to the number of NOX-related sequences identified by the authors) that we have added to the existing eukaryotic NOX family and have used to build an expanded phylogenetic tree. We cloned and overexpressed the identified *nox* gene from *Streptococcus pneumoniae* and confirmed that it codes for an NADPH oxidase. The membrane of the *S. pneumoniae* NOX protein (SpNOX) shares many properties with its eukaryotic counterparts, such as affinity for NADPH and flavin adenine dinucleotide, superoxide dismutase and diphenylene iodonium inhibition, cyanide resistance, oxygen consumption, and superoxide production. Traditionally, NOX enzymes in eukaryotes are related to functions linked to multicellularity. Thus, the discovery of a large family of NOX-related enzymes in the bacterial world brings up fascinating questions regarding their role in this new biological context.

## IMPORTANCE

NADPH oxidase (NOX) enzymes have not yet been reported in bacteria. Here, we carried out computational and experimental studies to provide the first characterization of a prokaryotic NOX. Out of 996 prokaryotic proteins showing NOX signatures, we initially selected, cloned, and overexpressed four of them. Subsequently, and based on preliminary testing, we concentrated our efforts on *Streptococcus* SpNOX, which shares many biochemical characteristics with NOX2, the referent model of NOX enzymes. Our work makes possible, for the first time, the study of pure forms of this important family of enzymes, allowing for biophysical and molecular characterization in an unprecedented way. Similar advances regarding other membrane protein families have led to new structures, further mechanistic studies, and the improvement of inhibitors. In addition, biological functions of these newly described bacterial enzymes will be certainly discovered in the near future.

## INTRODUCTION

Oxidative stress occurs when the balance between the generation of free radicals and their removal by antioxidants is lost. Free radicals are normally generated in the human body by metabolic processes, the best known being superoxide, peroxide, and radical hydroxyl oxygen species, which are collectively called reactive oxygen species (ROS). These species, which are highly reactive and can attack proteins, lipids, and nucleic acids, are major contributors to damage in biological organisms (1). These damages include aging-associated pathologies, cardiovascular disease, neurological disorders, fatty liver disease, cancers, and chronic inflammation (2). However, it is also apparent that ROS play essential roles in normal physiological processes, such as redox regulation, brain physiology, blood pressure, cell differentiation, hormone synthesis, and the very well-documented host defense system (3, 4). ROS-producing systems include xanthine oxidase, the mitochondrial respiratory chain, peroxidases, cytochrome P450 enzymes, uncoupled endothelial NO synthase, and phagocytic NADPH oxidases (NOXs). NOX proteins were the first identified systems that generate ROS not as a by-product but rather in a dedicated and targeted manner.

**FIG 1:**
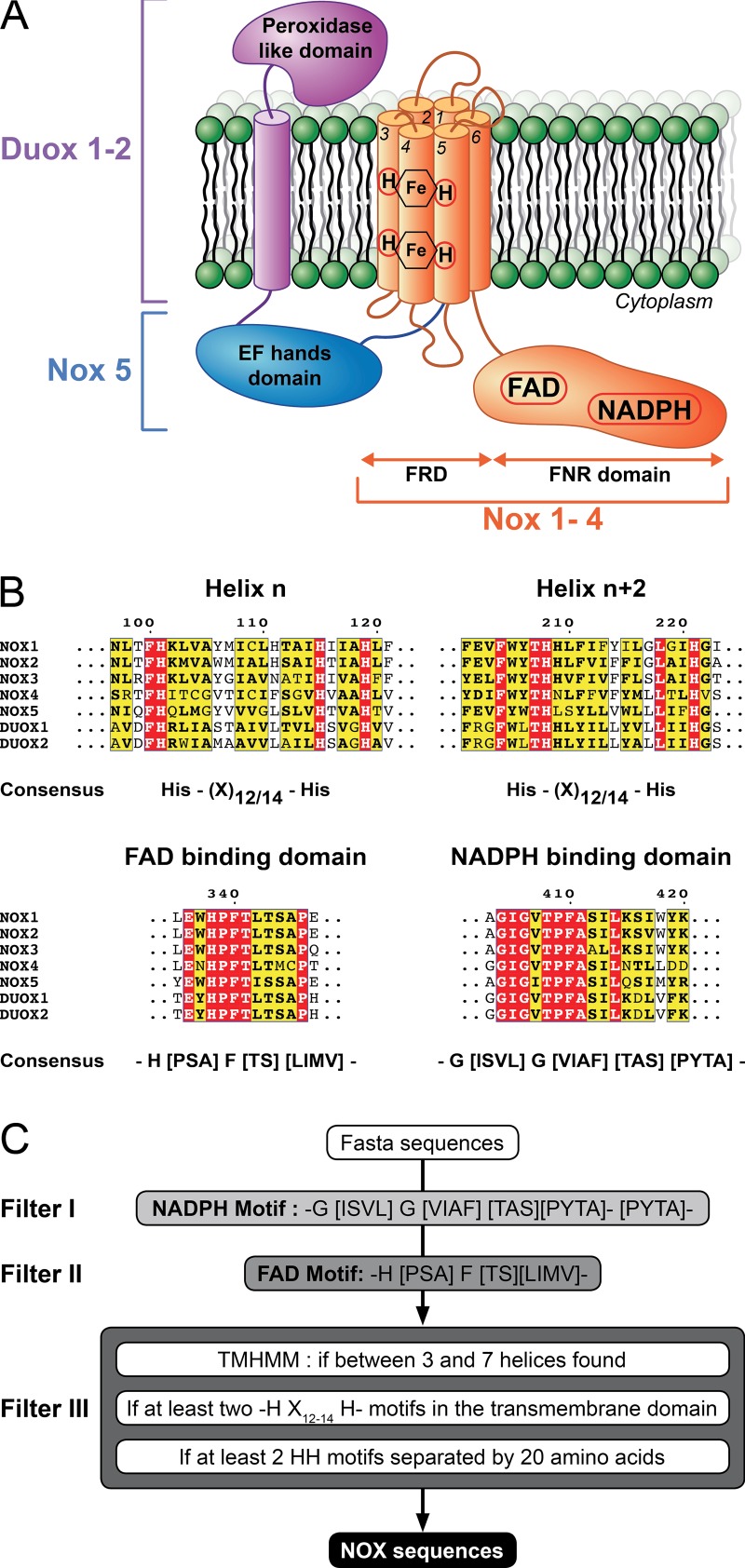
(A) Schematic representation of the NOX/DUOX protein family. (B) Multiple-sequence alignment of human NOX and DUOX proteins. Only the motifs used in this study are displayed. Amino acids strictly and partially conserved are highlighted in red and yellow, respectively. (C) Chart of the different filters used in this study to identify NOX sequences from Fasta formatted sequences. Each filter is independent and can be used separately.

Eukaryotic NOXs are generally known to catalyze one-electron transmembrane transfer to molecular oxygen, resulting in the synthesis of superoxide anion, the initial ROS. Historically, gp91^*phox*^, the NADPH oxidase from phagocytes and now called NOX2, was the first NOX described to produce ROS during the respiratory burst of the innate immunity process (5–10). Its first homolog was found in 1996 in rice (11), and within a few years five other NOXs were identified in mammals (6, 10, 12–17). The NOX family was subsequently divided into three subgroups, depending on the presence or absence of additional domains associated with the NOX2 protein core. They are widely distributed, tissue-specific modular proteins that share, for NOX1 to -4, a core consisting of a ferredoxin-NADP^+^ reductase (FNR)–like domain (9) and a membrane-bound ferric reductase-like domain (FRD) called the YedZ domain in prokaryotes (18, 19) (Fig. 1A). They may also have, like NOX5, EF-hand domains and, in addition, as for dual oxidase 1 (DUOX1) and DUOX2, an amino-terminal peroxidase homology segment (3) (Fig. 1A). Besides NOX2, which is found in phagocytes, cardiac tissue and brain, where it is involved in host defense, muscle contraction, and neural degeneration, respectively (20), NOX enzymes from nonimmune tissues are involved in processes such as cell proliferation, apoptosis, and receptor signaling (3). Conversely, DUOX enzymes are implicated in reactions that involve extracellular matrix proteins, such as reactions for thyroid hormone synthesis (21, 22).

Blocking the undesirable actions of NOX enzymes is a strategy for treating oxidative stress-related pathologies and damage. In addition to ROS-provoked damage, deregulation of NOX-dependent ROS production can have pathological consequences related to a specific physiological context (e.g., chronic granulomatous disease, autoimmune disorders, hypothyroidism when ROS production is impaired, or cardiovascular and neurodegenerative diseases in the case of ROS overproduction) (21, 23).

Traditionally, the presence of NOX enzymes has been linked to functional requirements related to multicellularity (24). An exception is the yeasts *Saccharomyces cerevisiae* and *Schizosaccharomyces pombe*, which are unicellular organisms known to contain open reading frames (ORFs) homologous to NOX in its genome. However, except in one case (25), these ORFs have not been described as NOXs but as ferric reductases (FRE) (26). FRE and NOX enzymes have different final electron acceptors, O_2_- and Fe^3+^-chelating complexes, respectively, which are attributed to a few amino acid sequence changes within their FRD. Nevertheless, they have been traditionally placed within the same global protein family in phylogenetic analyses (18, 27). Although both FNR and YedZ, the model of bacterial FRD protein recently renamed MsrQ (18, 19, 28), are found separately in prokaryotes, the presence of functional *nox* genes in bacterial genomes containing both domains has not been established (29).

## RESULTS

### Computational screening of putative NOX sequences.

**FIG 2:**
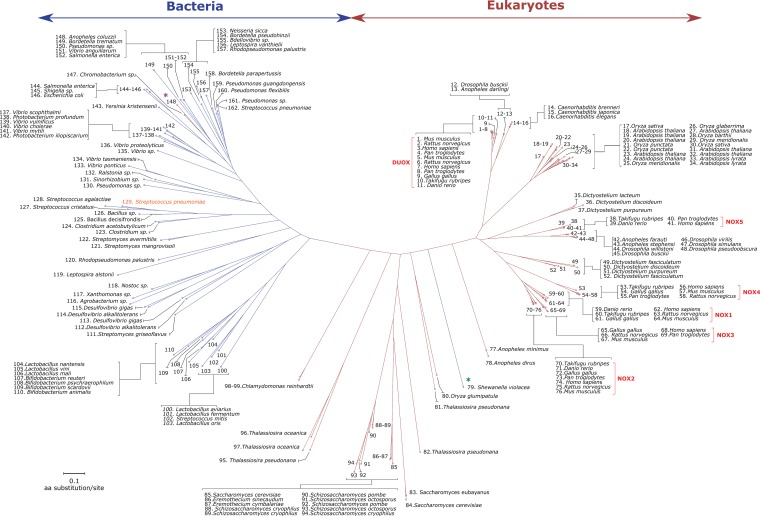
Phylogenetic tree of the NOX family. The tree was constructed using 162 sequences out of the 4,404 sequences identified in this study, and the sequences came from genera representative of all life domains (56). Eukaryote and prokaryote sequences are shown in red and blue, respectively. The green star highlights the only prokaryote sequence present in a eukaryote branch of the tree. The purple star highlights the unique eukaryote sequence present in the prokaryote branch of the tree. Each subfamily is indicated by blue square brackets. SpNOX is displayed in orange.

An algorithm was devised to search for bacterial NOX sequences, using only the following essential eukaryotic motifs: (i) NADPH- and FAD-binding domains, both NOX specific, and (ii) two heme-binding His-X_12/14_-His sequences (Fig. 1B). These filters were introduced to keep only sequences of proteins of interest for the next step. The final filter included the selection of *bis*-hystidyl patterns separated by at least 20 amino acids, which is the average length of a transmembrane α-helix, as well as a predicted transmembrane domain with at least three α-helices (Fig. 1C). The latter condition was used because the cytochrome *b*-like domain, which consists of six α-helices, is expected to bind the two-heme moieties at helices number 3 and 5. Starting from the 78,037,012 ORFs found in the UniprotKB/Swiss-Prot and UniProt/TrEMBL databases, our algorithm selected 4,404 putative NOX amino acid sequences from 1,492 different organisms, over a third of which were bacteria (Table 1). Conversely, only one archaeal sequence was found that fulfilled all of the above selection criteria (Fig. 1C). Subsequently, we selected a smaller set of sequences from all the represented domains of life (see Table S2 in the supplemental material) by removing (i) sequences from related organisms with >99% homology and (ii) a few sequences where the above motifs were not correctly aligned. Finally, 162 ORFs were selected to build an extended phylogenetic tree for the NOX/FRE family (see Table S3 in the supplemental material). The tree (Fig. 2) shows a clear division between eukaryotes and prokaryotes, which may have evolutionary significance.

**TABLE 1:** Statistical analysis results from the search for NOX sequences in the UniProt database^a^

Data set	No. of sequences		
	Swiss-Prot	TrEMBL	Total
Total	553,474	77,483,538	78,037,012
Filter I (NADPH)	575	252,407	253,164
Filter II (FAD)	64	7,375	7,439
Filter III (HH)	47	4,357	4,404
Eukaryotes	47	3,354	3,401
Prokaryotes	0	996	996
Archaea	0	1	1
Unclassified	0	6	6

^a^ The table shows the total number of sequences in the Swiss-Prot and TrEMBL databases, the number of sequences selected by the software after each filtering step, the number of eukaryotic, prokaryotic, and archaea sequences in each data set.

### Selection of membrane protein candidates from bacterial genomes.

In order to confirm that the sequences we extracted from bacterial genomes corresponded to functional NOX enzymes, we selected genes from *Acaryochloris marina*, *Escherichia coli*, *Pseudomonas aeruginosa*, and *Streptococcus pneumoniae* (AmNOX, EcNOX, PaNOX, and SpNOX, respectively) for further characterization. Their predicted protein topologies are very similar to the one calculated for NOX2, except for AmNOX, which is closer to NOX5 (Fig. S1). The selected genes were cloned and overexpressed in *E. coli* (Fig. S2), and the determination of the integrity of the resulting proteins was followed by spectrophotometry of the membrane extracts. Indeed, eukaryotic NOX enzymes are known to have a spectral signature with characteristic peaks at 426 nm and 558 nm in a difference (reduced minus oxidized) spectrum. The negative control was the same *E. coli* strain transformed by an expression plasmid devoid of a bacterial putative NOX gene. SpNOX, followed by PaNOX, were the proteins that showed the stronger spectroscopic signals (Fig. S3). Based on these results, we decided to pursue our study with SpNOX.

10.1128/mBio.01487-17.1 FIG S1  The predicted topologies of NOX2, SpNOX, EcNOX, PaNOX, and AmNOX consist of six transmembrane helices with 4 conserved histidines (red circles) in the transmembrane domain, a NADPH-binding domain (purple circles), and a FAD-binding domain (green circles). Schemes were prepared using the Protter server. Download FIG S1, JPG file, 2.7 MB. Copyright © 2017 Hajjar et al. 2017 Hajjar et al. This content is distributed under the terms of the Creative Commons Attribution 4.0 International license.

10.1128/mBio.01487-17.2 FIG S2  SDS-PAGE expression and Western blotting results (using an anti-His tag antibody) for four prokaryotic NOXs (see the main text for abbreviations). Four samples of each construct were characterized via SDS-PAGE prior to IPTG induction (*t* = 0) or after 1 h of induction (*t* = 1), 2 h of induction (*t* = 2), and 3 h of induction (*t* = 3). The black dot indicates the different expressed proteins. (Bottom panel) The Western blot results showed an expression leakage before induction. The problem was fixed with an *E. coli* strain containing a pLysS vector. Download FIG S2, JPG file, 2 MB. Copyright © 2017 Hajjar et al. 2017 Hajjar et al. This content is distributed under the terms of the Creative Commons Attribution 4.0 International license.

10.1128/mBio.01487-17.3 FIG S3  Spectral characterization of membrane preparations from *E. coli* expressing SpNOX (red), PaNOX (blue), EcNOX (green), and AmNOX (orange). The control spectrum (gray) was obtained using an *E. coli* membrane preparation transformed with an expression vector devoid of the bacterial NOX genes. Download FIG S3, JPG file, 0.7 MB. Copyright © 2017 Hajjar et al. 2017 Hajjar et al. This content is distributed under the terms of the Creative Commons Attribution 4.0 International license.

### SpNOX exhibits a cytochrome *b*_558_ spectrum typical of NOX enzymes.

**FIG 3:**
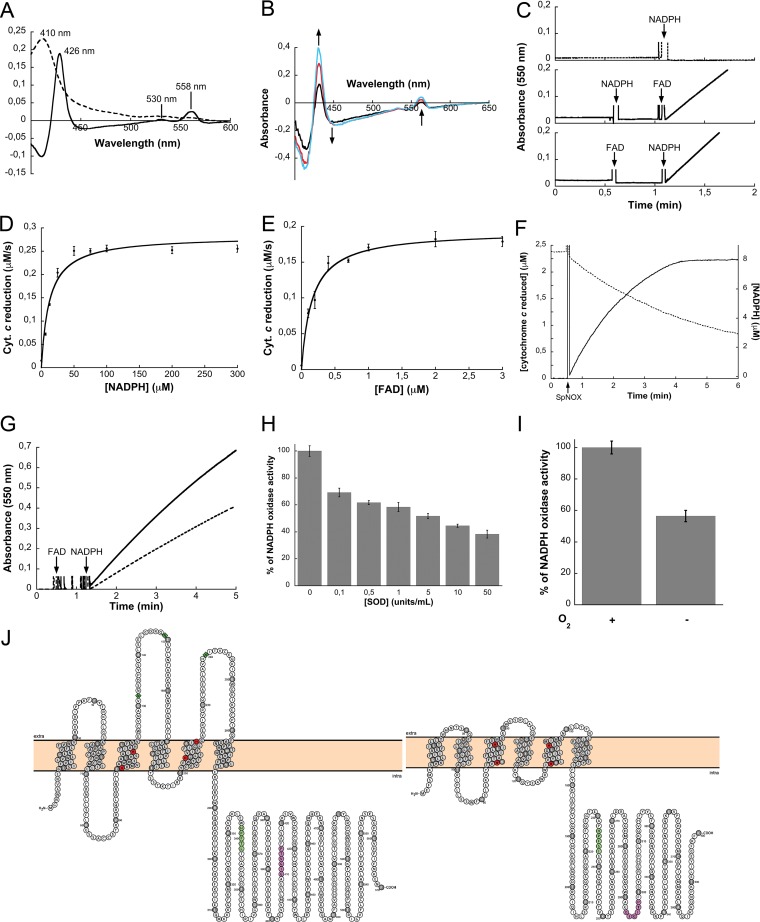
Enzymatic characterization of SpNOX. (A) Difference (reduced minus oxidized; solid line) and oxidized (dashed line) spectra from an *E. coli* membrane preparation expressing SpNOX. The typical peaks of NOX proteins are highlighted. (B) Absorbance spectra of a membrane preparation expressing SpNOX in the presence of 200 µM NADPH. Membrane reduction was sequentially recorded after 2 min (black line), 6 min (red line), and 10 min (blue line). Heme reduction was followed by an increase in peak height at 426 nm and 558 nm, showing that electrons can reach the membrane-bound core of SpNOX. (C) The activity of pure SpNOX was monitored in the presence or the absence of 2 µM FAD. (D and E) Michaelis-Menten saturation curves of SpNOX activity as a function of either NADPH concentration in the presence of 10 µM FAD (D) or FAD concentration in the presence of 200 µM NADPH (E). (F) NADPH oxidation (dashed line) and cytochrome *c* reduction (solid line) were followed simultaneously at 340 nm and 550 nm, respectively. (G) The reduction of cytochrome *c* (bold line) was inhibited by the addition of 0.3 mg/ml of SOD. (H) Inhibition effects of increasing SOD concentrations on SpNOX NADPH oxidase activity (*n* = 5). (I) SpNOX NADPH activity in either the presence (+) or absence (−) of O_2_; this experiment was performed under argon. (J) The predicted NOX2 and SpNOX topologies consist of six transmembrane helices with 4 conserved histidines (red circles) in the transmembrane domain, NADPH-binding site (green circles), and FAD-binding site (purple circles). The NOX2 glycosylation sites are shown as green squares. Schemes were prepared using the Protter server (http://wlab.ethz.ch/protter/start/) and the topologies were predicted by the TMHMM server (http://www.cbs.dtu.dk/services/TMHMM/).

SpNOX contained in the membrane preparation generated a spectrum similar to the one from NOX2. The dithionite-reduced difference spectrum showed a small peak at 558 nm, a shoulder at 530 nm, and a sharp peak at 426 nm (Fig. 3A) (30). As with many other cytochromes, the oxidized SpNOX gave a spectrum with a characteristic Soret band at 410 nm. The reduction of the heme *b* groups was followed by spectrophotometry, after the addition of NADPH. Increasing incubation times resulted in more pronounced peaks at 426 nm and 558 nm, while the spectrum decreased at 450 nm. These spectral changes indicate flavin reduction after electron transfer from NADPH (Fig. 3B) (31). Furthermore, heme reduction showed that electrons migrated from NADPH to the heme groups through the flavin cofactor, which is necessary to uncouple them (Fig. 3B).

### SpNOX and NOX2 share enzymatic properties.

A detergent screening was performed to identify the best-suited detergent to solubilize and stabilize SpNOX in an active form. The detergents of the maltoside family fulfilled all these requirements (Fig. S4). The His-tagged protein was found to be pure after its passage through a nickel affinity column followed by a size exclusion chromatographic step (Fig. S5). Next, the NADPH oxidase activity of the purified SpNOX was measured. It is well known that FAD is a required cofactor for the activity in eukaryotic NOX enzymes. Accordingly, the activity assay was performed with and without FAD, and superoxide production was determined by using a reporting system based on cytochrome *c* reduction. We found that purified SpNOX activity was dependent on FAD supplementation (Fig. 3C). However, the kinetic activity was strictly proportional to the final protein concentration, confirming that this activity is generated by an enzymatic process (Fig. S6A and B). SpNOX has lower affinities for NADPH and FAD (*K*_*m*_ of 8 µM and 0.13 µM) than NOX2 (*K*_*m*_ of 25 to 50 µM and 1 nM) (32, 33) (Fig. 3D and E) (34). It also exhibited a specific activity of 19 mol O_2_^•−^/mol SpNOX/s, which represents 30% of that one reported for NOX2 (which is 64 mol O_2_^•−^/mol cytochrome *b*/s [35]). Conversely, SpNOX has about twice the estimated specific activity of NOX4 (36). The stoichiometry of the reaction was established by simultaneously following substrate oxidation and cytochrome *c* reduction (Fig. 3F). One NADPH molecule should be able to transfer two electrons, ultimately producing two superoxide ions. From the kinetics presented in Fig. 3F, we obtained between 1.4 and 1.6 reduced cytochrome *c* molecules per oxidized NADPH molecule. This ratio compared well with the theoretical maximum value of 2, with the deficit explained by incomplete O_2_^•−^ scavenging by cytochrome *c* (37, 38). Moreover, replacement of His heme ligands in the H69A-H129A and H83A-H142A variants resulted in membrane fractions displaying the same basal activity level as the membrane fraction of *E. coli* transformed with the empty expression vector (Fig. S6C and D). Thus, the NADPH oxidase activity observed in SpNOX depends on electron transfer through the hemes and, consequently, it is not due to electron loss at the SpNOX FNR domain.

10.1128/mBio.01487-17.4 FIG S4  Spectroscopic results for protein stability using different detergents. The spectral signature of the heme cofactor was used as a reporter of protein folding. The critical micellar concentrations (CMC) of +5 mM (solid line) and +20 mM (dashed line) were tested. The best detergents were DDM and Cymal 7. Unfortunately, the latter inhibited SpNOX activity. Subsequently, we used DDM and, finally, its dimeric version, MNG3, which gave the best results. Download FIG S4, JPG file, 1 MB. Copyright © 2017 Hajjar et al. 2017 Hajjar et al. This content is distributed under the terms of the Creative Commons Attribution 4.0 International license.

10.1128/mBio.01487-17.5 FIG S5  Purification of SpNOX. The elution profile shows a symmetrical SpNOX peak after the last purification step (Superdex 200 gel filtration). The SDS-PAGE analysis (bottom inset) showed bands corresponding to the membrane fraction before solubilization (Mb), the sample loaded onto the nickel column (L), the flowthrough solution (FT), the washing solution (W) of the affinity step, and the protein that eluted from the size exclusion column. The affinity and size exclusion steps were coupled together, so only the samples before (L) and after (E) the purification procedure were analyzable. The presence of the heme cofactors in SpNOX produced a reddish color for the protein sample, which helped us follow it during purification. The purified protein sample was homogeneous, as confirmed by mass spectrometry. Download FIG S5, JPG file, 1.1 MB. Copyright © 2017 Hajjar et al. 2017 Hajjar et al. This content is distributed under the terms of the Creative Commons Attribution 4.0 International license.

10.1128/mBio.01487-17.6 FIG S6  Supplemental enzymatic characterization. (A) The kinetic activity was followed at 550 nm at different final protein concentrations. (B) The initial velocities are plotted on the right side of the figure. The curve could be fit to a straight line. Each condition was assayed at least 3 times. (C) The NADPH oxidase activity assay was performed with the SpNOX with the histidine ligands of the hemes mutated to alanine (see main text). The activity assays were performed with membrane fractions overexpressing the mutants and were compared to the wild-type protein activity. (D) The expression of the mutants was checked by Western blotting. The quadruplet mutant (Q) is labeled. Download FIG S6, JPG file, 1.5 MB. Copyright © 2017 Hajjar et al. 2017 Hajjar et al. This content is distributed under the terms of the Creative Commons Attribution 4.0 International license.

### A superoxide dismutation assay showed O_2_^•−^ production by SpNOX.

Superoxide production was also demonstrated by addition of superoxide dismutase (SOD) to the reaction mixture, which decreased cytochrome *c* reduction kinetics to 60% (Fig. 3G and H). The remaining SOD-insensitive activity (40%) likely resulted from direct cytochrome *c* reduction by SpNOX. Indeed, similar assays carried out under an argon atmosphere confirmed the direct electron transfer from SpNOX to cytochrome *c*, albeit with lower efficiency (Fig. 3I). This result can be rationalized by comparing the topologies of NOX2 and SpNOX. The extracellular A, C, and E loops are significantly shorter in SpNOX than in NOX2. In the context of this *in vitro* assay, this topology difference may facilitate the access of cytochrome *c* to the external heme of SpNOX, closer to the extracellular side (Fig. 3J). Another possibility is the direct reduction by FAD, because both sides of SpNOX are accessible in the detergent-solubilized form of the protein.

### Diphenylene iodonium inhibits SpNOX.

**FIG 4:**
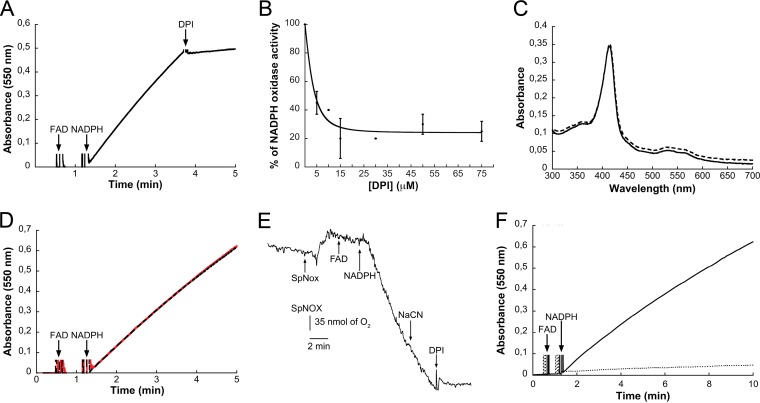
NOX activity of SpNOX. (A) SpNOX activities were measured after adding NADPH and FAD to final concentrations of 200 µM and 10 µM, respectively. The enzymatic activity was inhibited by the addition of 50 µM DPI (arrow). (B) Percentage of NADPH oxidase activity inhibition when monitored at different DPI concentrations. The error bars correspond to the standard deviations (*n* = 3). (C and D) Soret peak-containing spectra of SpNOX before (solid line) and after (dashed line) cyanide addition. As with eukaryotic NOXs, cyanide had no effect on the enzymatic activity of SpNOX. (E) Kinetics of cyanide-insensitive oxygen consumption by SpNOX. Only DPI inhibited this process. (F) The reduction of the ferric iron (dashed line) was inhibited by the addition of 0.4 mg/ml of SOD.

DPI inhibits flavoenzymes such as NOX2 (36) and is widely used as a common inhibitor of NOX enzymes. The effect of DPI (39, 57) on SpNOX activity was evaluated in the cytochrome *c* reduction assay. Addition of DPI immediately stopped the reaction (Fig. 4A). This inhibitor reacts with reduced FAD to form a phenyl radical, which can either attack amino acids found at or near the FAD-binding site or directly attack the cofactor, ultimately resulting in irreversible enzyme inhibition (40). The calculated DPI 50% inhibitory concentration was around 5 µM for 1 µM FAD in our assay (Fig. 4B).

### Cyanide does not inhibit SpNOX activity.

Cyanide is known to be a global inhibitor of hemoproteins, thanks to its axial binding to the iron ion. In contrast to this general rule, NOX enzymes are insensitive to both azide and cyanide; this feature represents a hallmark of NOX proteins, where both hemes are expected to be hexa-coordinated (6). We have also assessed this property for SpNOX by using catalase as a positive control. While cyanide incubation induced a shift of the 417 nm Soret band of catalase (data not shown), no modification was observed in the SpNOX spectrum upon NaCN addition (Fig. 4C). Furthermore, its NADPH oxidase activity was identical with or without added cyanide or azide (Fig. 4D). Finally, SpNOX-dependent oxygen consumption, measured using a Clark electrode (31) (Fig. 4E), confirmed its insensitivity to NaCN addition. Taken together, these data show that electrons transferred from NADPH to the heme groups through FAD can use molecular oxygen as the final acceptor. This activity, along with the DPI sensitivity and cyanide resistance described above, is indicative of the structural and functional relatedness of SpNOX to the eukaryotic NOX family of NADPH oxidases.

### SpNOX is not a “true” ferric reductase.

Within the global NOX/FRE family of enzymes harboring FRD and FNR domains, a distinction based on the final electron acceptor is often made between NOX/DUOX enzymes, which are capable of using oxygen as the electron acceptor, and FRE enzymes, which directly reduce the iron-chelating complex. In order to characterize further our bacterial NOX-related enzyme, we tested whether, in addition to its capacity to use oxygen as described above, SpNOX also had direct ferric reductase activity, as defined in a classical assay. We found that SpNOX was not able to reduce Fe^3+^ to Fe^2+^ in the presence of SOD (Fig. 4F). This showed that the observed ferric reductase activity of SpNOX is due to the produced superoxide ions that, in turn, are capable of reducing ferric iron. The specific activity was estimated to be 7.9 mol of Fe^3+^ reduced/mol of SpNOX/s, which represents 32% of NOX2’s ferric reductase activity. Indeed, the same experiment was performed using the human cytochrome *b*_558_, for which the specific activity was calculated to be 24 mol of Fe^3+^ reduced/mol of cytochrome *b*/s (data not shown). The SpNOX to NOX2 ratios of NADPH oxidase activity and ferric reductase activity were comparable, as expected from the dependency of the latter on the production of O_2_^•−^ by the former. Thus, within the NOX/FRE family of enzymes, SpNOX appears to be more related to a NOX than to a FRE, as indicated by its *in vitro* reactivity.

## DISCUSSION

Although new members of the NOX family of electron transfer membrane proteins have been continuously discovered since the 2000s, they have been restricted to eukaryotic organisms, from fungi to animals and plants. In 2004, a first indication of an evolutionary relationship of NOX enzymes with prokaryotic proteins was suggested by the simultaneous description by Von Rozycki et al. (19) and Sanchez-Pulido et al. (27) of the bacterial MsrQ protein. MsrQ was described as an FRD involved in electron transfer and significantly homologous to the membranous moiety of eukaryotic NOX proteins. Along the same lines, we reported recently that the flavin reductase Fre could be the potential electron donor to MsrQ. Thus, these two proteins mimic the architectural organization of eukaryotic NOX (38). This may be considered an intermediate evolutionary organization, ancestral to the eukaryotic NOX, where FRD and FNR domains are fused into a single polypeptide chain (38). The work presented here goes far beyond preliminary reports (19, 27), because we provide the first evidence for the existence of a large family of prokaryotic membrane proteins that have all the sequence motifs and predicted topological characteristics of NOX-related enzymes. All these prokaryotic sequences are well separated from their eukaryotic counterparts in the phylogenetic tree (Fig. 2). This suggests that they constitute a new subgroup in the NOX family of enzymes. A few bacterial sequences are located in the eukaryotic branches of the tree. These NOX5-like sequences, found, for instance, in *Acaryochloris marina*, were probably horizontally transferred from eukaryotic genomes (18). Moreover, there is one eukaryotic sequence from *Anapheles coluzzii* present in the middle of the bacteria subgroup. This sequence is very close to that in *Chromobacterium* sp., which is a bacterium that can effectively colonize the intestines of *A. coluzzii* (41). The horizontal gene transfer between bacteria and animals is still controversial and in this particular case it could have resulted from contamination during genome sequencing (42, 43). Thus, with the combinatorial use of specific sequence motifs characteristic of NOX enzymes, we have been able to identify a large number of bacterial sequences having (i) six transmembrane helices with (ii) conserved bis-histidyl motifs within the third and fifth transmembrane helices and (iii) a C-terminal dehydrogenase region containing FAD- and NADPH-binding domains.

This bioinformatics study was complemented with a biochemical characterization of the representative bacterial SpNOX, which fully confirmed its close functional and structural relationship with the eukaryotic NOX enzymes. Indeed, both systems are able to use NADPH as an electron donor and oxygen as the final acceptor. As expected, we also found that electron transfer from NADPH to O_2_ requires FAD and heme cofactors. Furthermore, like well-described NOX proteins, SpNOX is both sensitive to the DPI inhibitor, which targets the FAD moiety, and insensitive to cyanide.

In spite of the many common features, there are some clear differences between SpNOX and mammalian NOXs. Indeed, in contrast to NOX1 to -4, SpNOX does not need a p22^*phox*^-like subunit for proper membrane integration. Moreover, the purified recombinant form of the protein is constitutively active *in vitro*, without requiring cytosolic factors or an activation process. Also, SpNOX is active in a detergent-solubilized form, which is not the case for the mammalian NOX enzymes, which need to be reconstituted in a lipid bilayer (35, 44). Indeed, in order to be active, NOX2 needs to be reconstituted in a membrane forming a complex with cytosolic factors, lipid polar heads, and phosphatidylinositol, which is the template for p47^*phox*^ binding and global assembly (45). Being independent from such an assembly process, SpNOX is fully functional when inserted in a detergent micelle. Thus, all in all, we believe that SpNOX is a good model to study the activated state of a mammalian NOX2-like enzyme. The recombinant production of SpNOX will allow for novel biophysical and biochemical studies of NOX-related membrane-bound enzymes.

Importantly, the presence of these enzymes in prokaryotes raises the question of what could be their function. The recent emergence of ROS as signaling molecules in eukaryotes has led to the concept of ROS signaling within and between cells (4, 20). An example of cross-cell communication is given by the slime mold *Dictyostelium discoideum*. Although it grows as a single amoeba when food is available, when starved the single cells aggregate, constituting a multicellular organism of up to a million amoebae, which forms a fruiting body that sporulates (46). The aggregate does not form if O_2_^•−^ is scavenged either pharmacologically or by overexpression of SOD (46). Bloomfield et al. suggested that this (O_2_^•−^)-based signaling mechanism arose early in the evolution of multicellular organisms and most likely relies on NOXs, which genes are present in *Dictyostelium* (14) (Fig. 2). Indeed, Lardy et al. demonstrated the central role of NOX homologs in different phases of development of the slime mold (47).

Until now, it has been accepted that bacterial ROS production depends on reactions that are not primarily intended for that purpose. This apparent opportunistic, or even accidental, use of ROS appears to be in stark contrast with the regulated O_2_^•−^ production by NOX and its possible transformation to H_2_O_2_ by SOD found in eukaryotes (2). Indeed, regulated ROS production is expected to be amenable to modulate intercellular signaling in a well-defined spatiotemporal context. In this context, our discovery that NOX-related enzymes are also present in bacteria (Fig. 2) is especially intriguing. Although, as far as we can tell, highly regulated generation of ROS in these microorganisms has not been reported, our results suggest that such process could be biologically important, for instance, for biofilm formation and dynamics. Indeed, bacterial biofilms, which are now known to be the most common way for bacteria to thrive in nature, have been considered an example of multicellularity (48). The use of NOX by bacterial communities could be a means to communicate within biofilms, as occurs in eukaryotes when O_2_^•−^ is generated in processes such as protozoan cell aggregation or to mediate, along with H_2_O_2_, the many other ROS-dependent signaling processes known to take place in metazoans.

An alternative possibility is that, although our bioinformatics and biochemical analyses indicated that SpNOX is a bona fide “NADPH oxidase,” it may not necessarily function, in its bacterial physiological context, as the eukaryotic NOXs do. For instance, bacterial NOX-like proteins may be components of electron transfer pathways where O_2_ is not the physiological electron acceptor. A recent example of such a possibility is the discovery that MsrQ transfers its electrons to MsrP, a molybdopterin protein acceptor present in the periplasm of *E. coli*, to catalyze the reduction of methionine sulfoxide in oxidatively damaged periplasmic proteins (49). The observation that SpNOX can directly reduce cytochrome *c* demonstrates that its more exposed heme is accessible to potential periplasmic redox partners. This provides some support to the hypothesis of alternative electron acceptors for bacterial NOX proteins in general. We conclude that many new and unanticipated functions and synthetic pathways may turn out to be associated with this new family of bacterial NOX.

An SpNOX knockout of *S. pneumoniae* did not display an identifiable phenotype (data not shown). It is clear that further studies will be required to address the role of bacterial NOX in signaling and/or metabolic processes, as postulated above.

In conclusion, the work reported here has allowed us to identify for the first time, both theoretically and functionally, a significant number of bacterial protein sequences that are found in current databases and are counterparts of eukaryotic NOX proteins.

## MATERIALS AND METHODS

### NOX sequence identification.

The software used to identify NOX sequences in bacterial genomes was written in Python 3.0. The program used three filters. The first two filters selected sequences containing H(PSA)F[TS][LIMV] (FAD-binding motif) and G(ISVL)G[VIAF][TAS][PYTA] (NADPH-binding motifs) (Fig. 1), defined using the 101 protein sequences identified by Kawahara et al. (50). The third filter selected sequences that contained the four histidine residues responsible for coordinating the two-heme moieties of the cytochrome *b*-like motif. The TMHMM sequence (51) was used to locate transmembrane α-helices. Sequences with more than seven predicted α-helices (the number found in DUOX1 and -2) were rejected, whereas sequences with at least 3 predicted α-helices were kept to account for errors in the prediction.

### Amino acid sequence alignments and phylogenetic tree construction.

Multiple sequence alignment of NOX sequences was carried out with Clustal Omega (52). The tree was built from 162 putative NOX sequences (Table S3) by using PhyML 3.0. The sequences used for software validation are provided in Table S1.

10.1128/mBio.01487-17.7 TABLE S1  Sequences used for software validation (for each organism, the total number of sequences used and those selected by the software after each filter are shown; the NOX column shows the number of sequences from filter III that are NOX proteins; only NOX proteins were selected by the software for all the validation tests). Download TABLE S1, DOCX file, 0.03 MB. Copyright © 2017 Hajjar et al. 2017 Hajjar et al. This content is distributed under the terms of the Creative Commons Attribution 4.0 International license.

10.1128/mBio.01487-17.8 TABLE S2  List of the genera used to build the phylogenetic tree shown in Fig. 2. Download TABLE S2, DOCX file, 0.03 MB. Copyright © 2017 Hajjar et al. 2017 Hajjar et al. This content is distributed under the terms of the Creative Commons Attribution 4.0 International license.

10.1128/mBio.01487-17.9 TABLE S3  NOX prokaryotic sequences used to build the phylogenetic tree. Download TABLE S3, DOCX file, 0.04 MB. Copyright © 2017 Hajjar et al. 2017 Hajjar et al. This content is distributed under the terms of the Creative Commons Attribution 4.0 International license.

### Cloning of the bacterial *nox* genes in a pQLink N vector.

Genes were optimized for expression in *E. coli* and synthetized via GenScript. They were inserted in a pUC57 vector conferring ampicillin resistance and providing a His tag coding sequence in frame with the 5′ end of the inserted gene. The pUC57 vectors containing the bacterial *nox* genes were treated with Not1 and BamHI restriction enzymes for 2 h at 37°C and ligated to a NotI-BamHI-digested pQLink N vector to yield pQLink N-bacterial NOX expression plasmids. Several expression strains were tested, and the best result was obtained with BL21(DE3) pLysS expression cells.

### SpNOX expression and purification.

The culture was grown in LB medium containing chloramphenicol (0.034mg/ml) and ampicillin (0.1 mg/ml) at 37°C with shaking. At an optical density at 600 nm of 1.5, isopropyl-β-d-thiogalactopyanoside (IPTG) was added to a final concentration of 0.1 mM with shaking at 210 rpm to induce the expression of the protein of interest. After 4 h of induction, cells were harvested by centrifugation (7,000 × *g*, 30 min, 4°C).

The culture pellet was resuspended in 50 mM Tris-HCl (pH 7), 250 mM NaCl, 10% glycerol supplemented with DNase and an antiprotease cocktail. Cells were disrupted using a microfluidizer (M-1105; Microfluidics, USA) at 1,400 lb/in^2^ (4 to 5 passes). Unbroken cells, debris, and inclusion bodies were removed by one low-speed centrifugation step at 8,000  × *g* in a Beckman rotor 850 followed by ultracentrifugation at 235,000  × *g* (Beckman Ti-45 rotor at 45,000 rpm) for 45 min at 4°C. The resulting pellet of membranes was solubilized to 2 mg/ml in 50 mM Tris-HCl (pH 7), 300 mM NaCl, and 5 mM (0.5%) lauryl maltose neopentyl glycol (NG310-MNG). Equilibrated Ni-nitrilotriacetic acid (NTA)–Sepharose resin was added to the mixture (1 ml per 15 mg of protein) and incubated overnight with shaking at 4°C.

Loaded resin was packed in a gravity column and washed thoroughly with Tris-HCl buffer (pH 7), 300 mM NaCl, 0.015% MNG, and 50 mM imidazole. The same buffer supplied with 300 mM imidazole was used to elute the protein. Pure fractions were concentrated and loaded on a Superdex 200 column previously equilibrated with Tris buffer (pH 7.0), 300 mM NaCl, and 0.003% MNG. The protein sample was stored at 4°C.

### Spectroscopy signatures.

Differential (reduced minus oxidized) spectra were recorded between 600 and 400 nm using a Cary 50 Bio UV/visible spectrophotometer (6). Reaction mixtures (total volume of 130 µl in quartz cuvettes) consisted of membranes at 1 mg/ml. The spectrum of reduced protein was obtained after adding a few crystals of sodium dithionite.

### NADPH oxidase activity assay.

NADPH oxidase activity was assayed by following the reduction of cytochrome *c* (58). NADPH and FAD were added to 0.1 µg of protein to final concentrations up to 200 µM and 10 µM, respectively; the extent of cytochrome *c* (100 µM) reduction was calculated based on the absorbance difference at 550 nm with an absorption coefficient of 21,000 M^−1^ cm^−1^. DPI was added to a final concentration of 50 µM. Adding 10 units of superoxide dismutase verified the specificity of the product.

### Measurement of cyanide-resistant oxygen consumption.

The oxygen consumption rate of 20 µg of purified SpNOX was measured by using a Clark electrode in a final volume of 1.6 ml. The oxygraph was calibrated by addition of dithionite to air-saturated buffer to establish the zero level of O_2_ concentration. Sodium cyanide and DPI were added at final concentrations of 1 mM and 50 µM, respectively (54).

### Ferric reductase activity assay.

Ferrous ions form a complex with ferrozine, which can be monitored at 562 nm. The Fe-NTA solution was prepared as described by Awai et al. (55). The ferrozine stock solution was prepared in 150 mM of acetate buffer (pH 7) to a final concentration of 100 mM. Ferric reduction was calculated based on the absorbance difference at 562 nm, using an absorption coefficient of 28,000 M^−1^ cm^−1^ (53). The reaction mixture consisted of 0.1 µg/ml SpNOX, 0.4 mM ferrozine, 0.3 mM Fe-NTA, and 10 µM FAD, with the addition of 200 µM NADPH to a final volume of 1 ml to start the reaction.
