# Supplementary figures and images for: The NOX Family of Proteins Is Also Present in Bacteria
Source: mBio. 2017 Nov 7;8(6):e01487-17. doi: 10.1128/mBio.01487-17 (PMC5676040; doi:10.1128/mBio.01487-17)

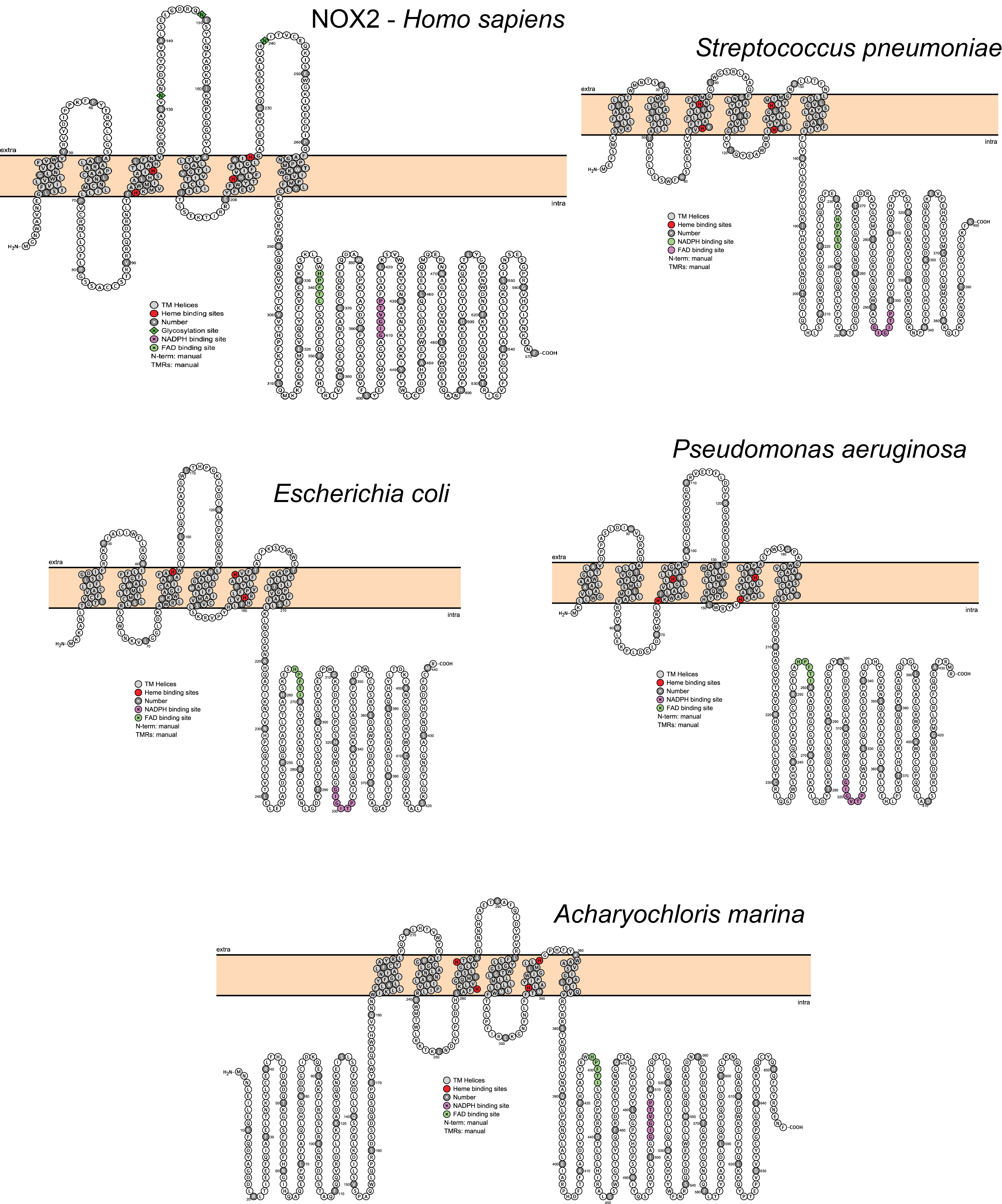

Supplement: FIG S1 [file mbo005173564sf1.jpg]

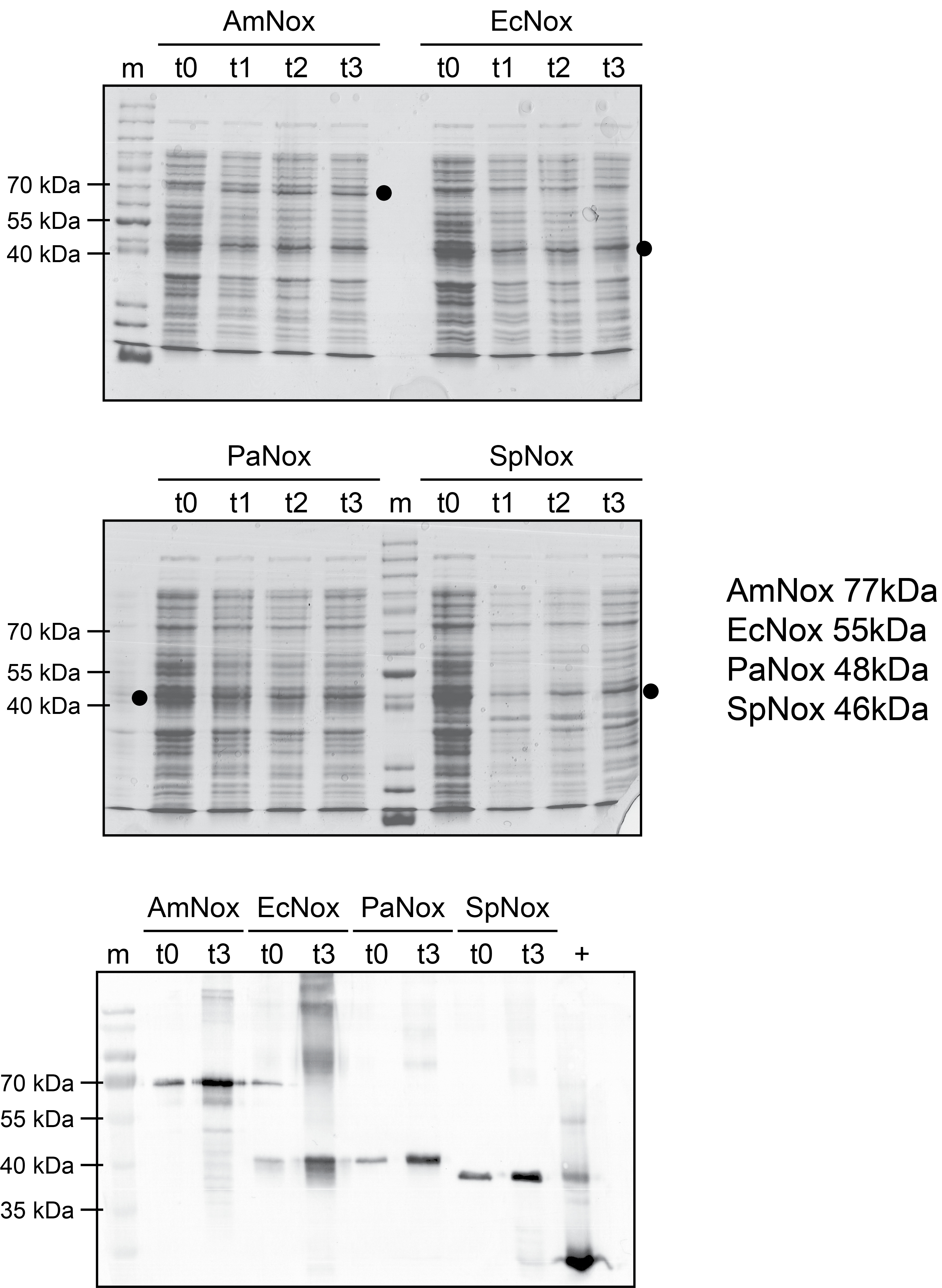

Supplement: FIG S2 [file mbo005173564sf2.jpg]

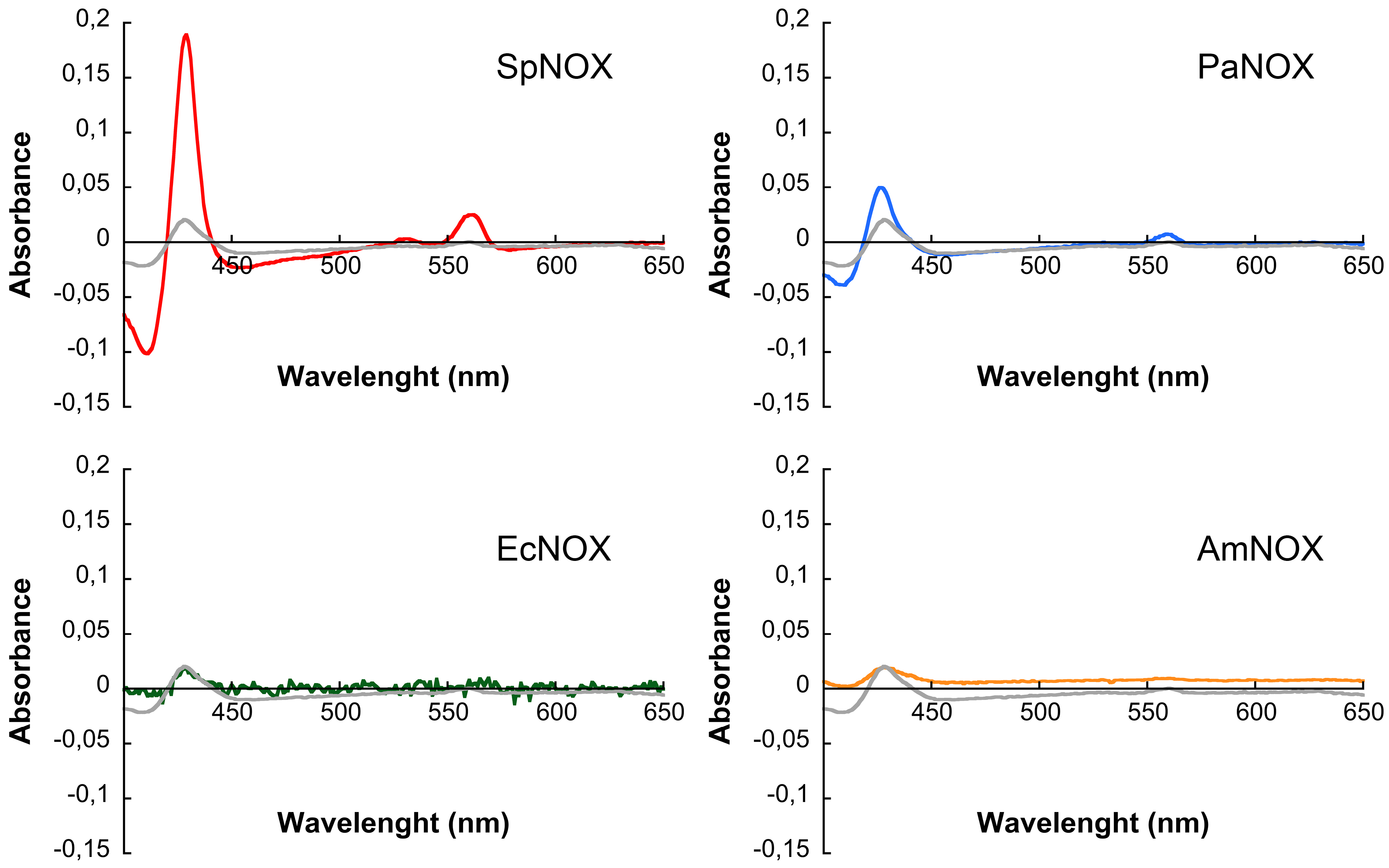

Supplement: FIG S3 [file mbo005173564sf3.jpg]

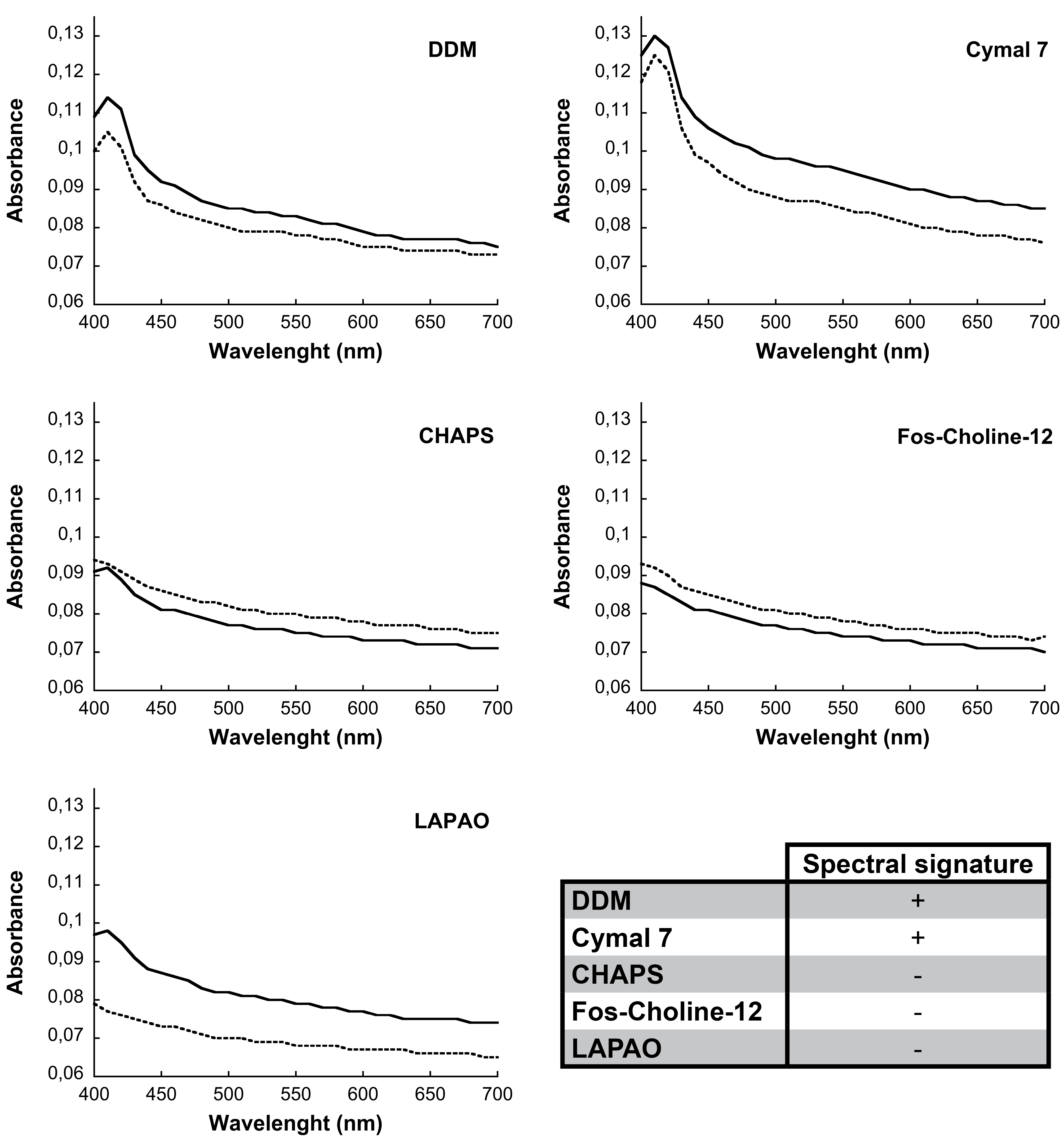

Supplement: FIG S4 [file mbo005173564sf4.jpg]

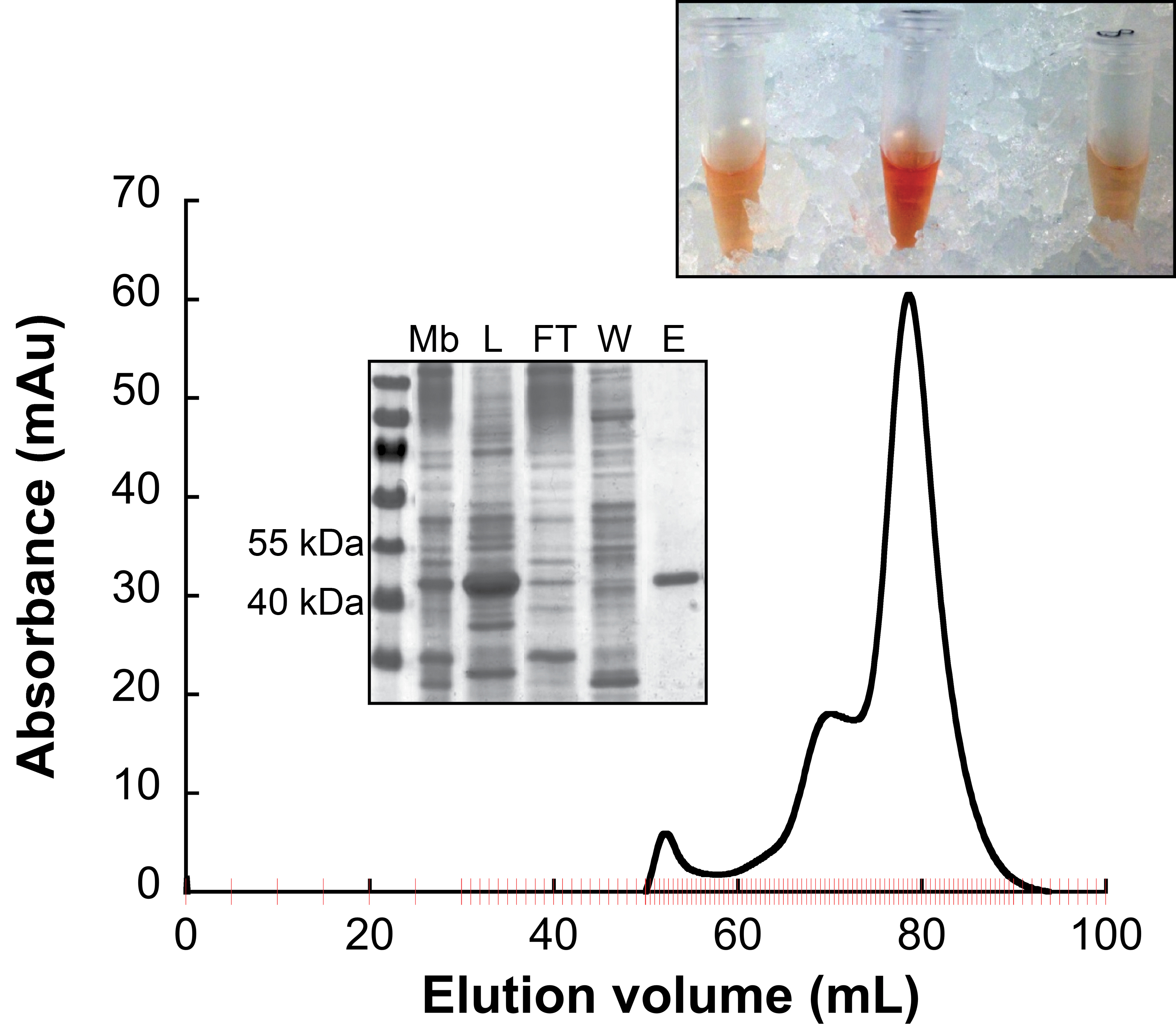

Supplement: FIG S5 [file mbo005173564sf5.jpg]

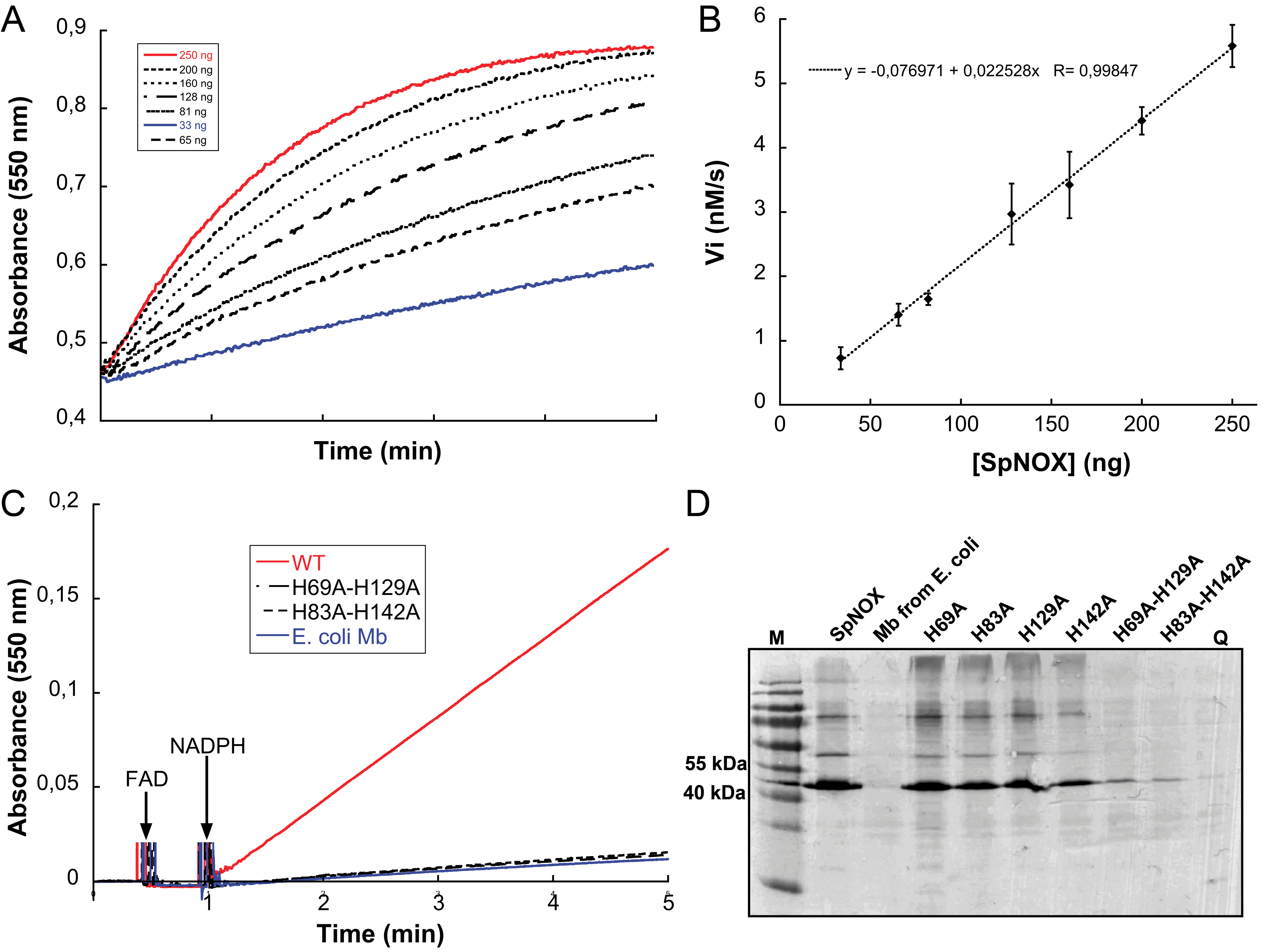

Supplement: FIG S6 [file mbo005173564sf6.jpg]
