# Supplementary material for: The NOX Family of Proteins Is Also Present in Bacteria
Source: mBio. 2017 Nov 7;8(6):e01487-17. doi: 10.1128/mBio.01487-17 (PMC5676040; doi:10.1128/mBio.01487-17)
Supplement: TABLE S1 [file mbo005173564st1.docx]

SUPPLEMENTARY TABLE 1:

| Organism | | Sequences | Step  I  (NADPH) | Step II  (FAD) | Step III  (HH) | NOX/FRE | | |
| --- | --- | --- | --- | --- | --- | --- | --- | --- |
|  |  |  |  |  |  | **TrEMBL** | **Swiss-Prot** | **Total** |
| *Homo sapiens* | Human | 131 321 | 543 | 28 | 21 | **13** | **8** | **21** |
| *Mus musculus* | Mouse | 75 665 | 536 | 18 | 12 | **8** | **4** | **12** |
| *Gallus gallus* | Chicken | 29 121 | 222 | 10 | 8 | **8** | **-** | **8** |
| *Xenopus tropicalis* | Frog | 30 522 | 233 | 9 | 8 | **8** | **-** | **8** |
| *Danio rerio* | Zebrafish | 57 186 | 342 | 13 | 10 | **10** | **-** | **10** |
| *Drosophila melanogaster* | Fly | 37 645 | 565 | 12 | 4 | **3** | **1** | **4** |
| *Arabidapsis taliana* | Mouse-ear cress | 54 227 | 374 | 20 | 11 | **1** | **10** | **11** |
| *Lotus japonicus* | Lotus corniculatus | 8 665 | 32 | 2 | 0 | **-** | **-** | **-** |
| *Zea mays* | Corn | 44 344 | 374 | 12 | 6 | **6** | **-** | **6** |
| *Chlamydomonas reinhardtii* | Green Algae | 15 118 | 547 | 6 | 2 | **2** | **-** | **2** |
| *Staurastrum punctulatum* | Green Algae | 110 | 1 | 1 | 0 | **-** | **-** | **-** |
| *Neurospora crassa* | Fungus | 13 326 | 169 | 3 | 1 | **1** | **-** | **1** |
| *Aspergillus nidulans* | Fungus | 13 291 | 156 | 3 | 2 | **2** | **-** | **2** |
| *Saccharomyces cerevisiae* | Yeast | 73 544 | 413 | 18 | 12 | **11** | **1** | **12** |
| *Candida albicans* | Yeast | 16 190 | 82 | 2 | 0 | **-** | **-** | **-** |
| *Acanthamoeba castellanii* | Amibia | 1 172 | 5 | 2 | 2 | **2** | **-** | **2** |
| *Tetrahymena thermophila* | Protozoa | 25 351 | 73 | 5 | 5 | **5** | **-** | **5** |
| *Trichomonas vaginalis* | Protozoa | 50 669 | 96 | 1 | 0 | **-** | **-** | **-** |
| *Giardia intertinalis* | Protozoa | 18 549 | 120 | 0 | 0 | **-** | **-** | **-** |
| *Bigelowiella natans* | Rhizaria | 575 | 2 | 0 | 0 | **-** | **-** | **-** |
