# Supplementary material for: The NOX Family of Proteins Is Also Present in Bacteria
Source: mBio. 2017 Nov 7;8(6):e01487-17. doi: 10.1128/mBio.01487-17 (PMC5676040; doi:10.1128/mBio.01487-17)
Supplement: TABLE S2 [file mbo005173564st2.docx]

SUPPLEMENTARY TABLE 2:

| Genus | Division |
| --- | --- |
| Aeropyrum | Archaea |
| Archaeoglobus | Archaea |
| Halobacterium | Archaea |
| Methanocaldococcus | Archaea |
| Methanococcus | Archaea |
| Methanopyrus | Archaea |
| Methanosarcina | Archaea |
| Methanothermobacter | Archaea |
| Nanoarchaeum | Archaea |
| Pyrobaculum | Archaea |
| Pyrococcus | Archaea |
| Sulfolobus | Archaea |
| Thermoplasma | Archaea |
| Acidobacterium | Eubacteria |
| Agrobacterium | Eubacteria |
| Aquifex | Eubacteria |
| Bacillus | Eubacteria |
| Bacteroides | Eubacteria |
| Bdellovibrio | Eubacteria |
| Bifidobacterium | Eubacteria |
| Bordetella | Eubacteria |
| Borrelia | Eubacteria |
| Bradyrhizobium | Eubacteria |
| Brucella | Eubacteria |
| Buchnera | Eubacteria |
| Campylobacter | Eubacteria |
| Caulobacter | Eubacteria |
| Chlamydia | Eubacteria |
| Chlamydophila | Eubacteria |
| Chlorobaculum | Eubacteria |
| Chromobacterium | Eubacteria |
| Clostridium | Eubacteria |
| Corynebacterium | Eubacteria |
| Coxiella | Eubacteria |
| Dehalococcoides | Eubacteria |
| Deinococcus | Eubacteria |
| Desulfovibrio | Eubacteria |
| Enterococcus | Eubacteria |
| Escherichia | Eubacteria |
| Fibrobacter | Eubacteria |
| Fusobacterium | Eubacteria |
| Gemmata | Eubacteria |
| Geobacter | Eubacteria |
| Haemophilus | Eubacteria |
| Helicobacter | Eubacteria |
| Lactobacillus | Eubacteria |
| Lactococcus | Eubacteria |
| Leptospira | Eubacteria |
| Listeria | Eubacteria |
| Mesorhizobium | Eubacteria |
| Mycobacterium | Eubacteria |
| Mycoplasma | Eubacteria |
| Neisseria | Eubacteria |
| Nitrosomonas | Eubacteria |
| Nostoc | Eubacteria |
| Oceanobacillus | Eubacteria |
| Onion | Eubacteria |
| Pasteurella | Eubacteria |
| Photobacterium | Eubacteria |
| Photorhabdus | Eubacteria |
| Pirellula | Eubacteria |
| Porphyromonas | Eubacteria |
| Prochlorococcus | Eubacteria |
| Pseudomonas | Eubacteria |
| Ralstonia | Eubacteria |
| Rhodopseudomonas | Eubacteria |
| Rickettsia | Eubacteria |
| Salmonella | Eubacteria |
| Shewanella | Eubacteria |
| Shigella | Eubacteria |
| Sinorhizobium | Eubacteria |
| Solibacter | Eubacteria |
| Staphylococcus | Eubacteria |
| Streptococcus | Eubacteria |
| Streptomyces | Eubacteria |
| Synechococcus | Eubacteria |
| Synechocystis | Eubacteria |
| Thermoanaerobacter | Eubacteria |
| Thermotoga | Eubacteria |
| Thermus | Eubacteria |
| Treponema | Eubacteria |
| Tropheryma | Eubacteria |
| Ureaplasma | Eubacteria |
| Vibrio | Eubacteria |
| Wigglesworthia | Eubacteria |
| Wolbachia | Eubacteria |
| Wolinella | Eubacteria |
| Xanthomonas | Eubacteria |
| Xylella | Eubacteria |
| Yersinia | Eubacteria |
| Anopheles | Invertebrates |
| Arabidopsis | Plants |
| Caenorhabditis | Invertebrates |
| Chlamydomonas | Green alga |
| Cryptosporidium | Invertebrates |
| Cyanidioschyzon | Red alga |
| Danio | Vertebrates |
| Dictyostelium | Invertebrates |
| Drosophila | Invertebrates |
| Eremothecium | Fungi |
| Gallus | Vertebrates |
| Giardia | Invertebrates |
| Homo | Primates |
| Leishmania | Invertebrates |
| Mus | Rodents |
| Oryza | Plants |
| Pan | Primates |
| Plasmodium | Invertebrates |
| Rattus | Rodents |
| Saccharomyces | Fungi |
| Schizosaccharomyces | Fungi |
| Takifugu | Vertebrates |
| Thalassiosira | Plants |
