# Supplementary material for: The NOX Family of Proteins Is Also Present in Bacteria
Source: mBio. 2017 Nov 7;8(6):e01487-17. doi: 10.1128/mBio.01487-17 (PMC5676040; doi:10.1128/mBio.01487-17)
Supplement: TABLE S3 [file mbo005173564st3.docx]

SUPPLEMENTARY TABLE 3:

| Sequence Uniprot ID | Organism species | Number in the phylogenic tree | Eucaryote | Procaryote |
| --- | --- | --- | --- | --- |
| A0A1B3NTL3 | Agrobacterium sp. RAC06 | 116 |  | X |
| A0A182KL84 | Anopheles coluzzii | 148 | X |  |
| W5J7V8 | Anopheles darlingi | 13 | X |  |
| A0A182N4G4 | Anopheles dirus | 78 | X |  |
| A0A182QCB6 | Anopheles farauti | 42 | X |  |
| A0A182VXX2 | Anopheles minimus | 77 | X |  |
| A0A182Y040 | Anopheles stephensi | 43 | X |  |
| D7MGJ6 | Arabidopsis lyrata subsp. lyrata | 33 | X |  |
| D7MQW8 | Arabidopsis lyrata subsp. lyrata | 34 | X |  |
| Q9FJD6 | Arabidopsis thaliana | 18 | X |  |
| Q9LZU9 | Arabidopsis thaliana | 19 | X |  |
| O81211 | Arabidopsis thaliana | 20 | X |  |
| Q9SUT8 | Arabidopsis thaliana | 23 | X |  |
| O48538 | Arabidopsis thaliana | 24 | X |  |
| Q9SBI0 | Arabidopsis thaliana | 27 | X |  |
| Q9FIJ0 | Arabidopsis thaliana | 31 | X |  |
| O81209 | Arabidopsis thaliana | 32 | X |  |
| A0A0M8PWZ9 | Bacillus decisifrondis | 125 |  | X |
| A3IFX7 | Bacillus sp. B14905 | 126 |  | X |
| A0A0B8WCB5 | Bdellovibrio sp. ArHS | 155 |  | X |
| D3R7Q6 | Bifidobacterium animalis subsp. lactis (strain BB-12) | 110 |  | X |
| A0A087CLQ5 | Bifidobacterium psychraerophilum | 108 |  | X |
| A0A087CM93 | Bifidobacterium reuteri DSM 23975 | 107 |  | X |
| A0A087D711 | Bifidobacterium scardovii | 109 |  | X |
| Q7W819 | Bordetella parapertussis (strain 12822 / ATCC BAA-587 / NCTC 13253) | 158 |  | X |
| A0A0J6C2N1 | Bordetella pseudohinzii | 154 |  | X |
| A0A157M3S9 | Bordetella trematum | 149 |  | X |
| G0MM94 | Caenorhabditis brenneri | 14 | X |  |
| O01795 | Caenorhabditis elegans | 16 | X |  |
| H2VZ19 | Caenorhabditis japonica | 15 | X |  |
| A8IRR2 | Chlamydomonas reinhardtii | 98 | X |  |
| A8IRR3 | Chlamydomonas reinhardtii | 99 | X |  |
| A0A0J6NCW0 | Chromobacterium sp. LK1 | 147 |  | X |
| Q97TQ5 | Clostridium acetobutylicum (strain ATCC 824 / DSM 792 / JCM 1419 / LMG 5710 / VKM B-1787) | 124 |  | X |
| K6TZ67 | Clostridium sp. Maddingley MBC34-26 | 123 |  | X |
| F1QVF2 | Danio rerio | 11 | X |  |
| E7F9M1 | Danio rerio | 39 | X |  |
| A7E3K4 | Danio rerio | 59 | X |  |
| Q7T2A7 | Danio rerio | 71 | X |  |
| S7UF45 | Desulfovibrio alkalitolerans DSM 16529 | 112 |  | X |
| S7TGC1 | Desulfovibrio alkalitolerans DSM 16529 | 114 |  | X |
| T2GDW1 | Desulfovibrio gigas DSM 1382 | 113 |  | X |
| T2GAN9 | Desulfovibrio gigas DSM 1382 | 115 |  | X |
| Q54F44 | Dictyostelium discoideum | 36 | X |  |
| Q86GL4 | Dictyostelium discoideum | 50 | X |  |
| F4QA30 | Dictyostelium fasciculatum (strain SH3) | 49 | X |  |
| F4QFB6 | Dictyostelium fasciculatum (strain SH3) | 52 | X |  |
| A0A151ZJZ5 | Dictyostelium lacteum | 35 | X |  |
| F1A617 | Dictyostelium purpureum | 37 | X |  |
| F0ZID3 | Dictyostelium purpureum | 51 | X |  |
| A0A0M4E2I1 | Drosophila busckii | 12 | X |  |
| A0A0M4EJJ3 | Drosophila busckii | 45 | X |  |
| Q291S3 | Drosophila pseudoobscura pseudoobscura | 48 | X |  |
| A0A0J9RE96 | Drosophila simulans | 47 | X |  |
| B4MEB4 | Drosophila virilis | 46 | X |  |
| B4MJV9 | Drosophila willistoni | 44 | X |  |
| G8JWZ2 | Eremothecium cymbalariae (strain CBS 270.75 / DBVPG 7215 / KCTC 17166 / NRRL Y-17582) | 87 | X |  |
| A0A0X8HS87 | Eremothecium sinecaudum | 86 | X |  |
| L2VL28 | Escherichia coli KTE10 | 146 |  | X |
| A0A1D5PJY4 | Gallus gallus | 9 | X |  |
| F1NWG5 | Gallus gallus | 54 | X |  |
| F1NEJ0 | Gallus gallus | 61 | X |  |
| A0A1D5PZP8 | Gallus gallus | 65 | X |  |
| A0A1D5P6A6 | Gallus gallus | 72 | X |  |
| Q9NRD9 | Homo sapiens | 3 | X |  |
| Q9NRD8 | Homo sapiens | 7 | X |  |
| Q96PH1 | Homo sapiens | 41 | X |  |
| B7Z523 | Homo sapiens | 56 | X |  |
| Q9Y5S8 | Homo sapiens | 62 | X |  |
| Q9HBY0 | Homo sapiens | 68 | X |  |
| P04839 | Homo sapiens | 74 | X |  |
| A0A0R1ZGB9 | Lactobacillus aviarius subsp. araffinosus DSM 20653 | 100 |  | X |
| C0WY04 | Lactobacillus fermentum ATCC 14931 | 101 |  | X |
| J1F5M4 | Lactobacillus mali KCTC 3596 | 106 |  | X |
| A0A0R1WHW0 | Lactobacillus nantensis DSM 16982 | 104 |  | X |
| A0A0R1WGC4 | Lactobacillus oris DSM 4864 | 103 |  | X |
| A0A0R2BU83 | Lactobacillus vini DSM 20605 | 105 |  | X |
| A0A1D7UXB0 | Leptospira alstonii | 119 |  | X |
| N1W143 | Leptospira vanthielii serovar Holland str. Waz Holland | 156 |  | X |
| A2AQ92 | Mus musculus | 1 | X |  |
| B7ZWM4 | Mus musculus | 5 | X |  |
| Q9JHI8 | Mus musculus | 57 | X |  |
| Q8CIZ9 | Mus musculus | 64 | X |  |
| Q672J9 | Mus musculus | 67 | X |  |
| Q61093 | Mus musculus | 76 | X |  |
| I2NW63 | Neisseria sicca VK64 | 153 |  | X |
| Q8YTT0 | Nostoc sp. (strain PCC 7120 / SAG 25.82 / UTEX 2576) | 118 |  | X |
| A0A0D3ENQ4 | Oryza barthii | 28 | X |  |
| I1NRG9 | Oryza glaberrima | 26 | X |  |
| A0A0D9ZLA3 | Oryza glumipatula | 80 | X |  |
| A0A0E0DU31 | Oryza meridionalis | 25 | X |  |
| A0A0E0FCY7 | Oryza meridionalis | 29 | X |  |
| A0A0E0LVI5 | Oryza punctata | 21 | X |  |
| A0A0E0M1N5 | Oryza punctata | 22 | X |  |
| B8BKW6 | Oryza sativa subsp. indica | 30 | X |  |
| B9G1I4 | Oryza sativa subsp. japonica | 17 | X |  |
| H2Q9C8 | Pan troglodytes | 4 | X |  |
| H2Q9C5 | Pan troglodytes | 8 | X |  |
| H2R8B3 | Pan troglodytes | 40 | X |  |
| H2R516 | Pan troglodytes | 55 | X |  |
| H2QTY3 | Pan troglodytes | 69 | X |  |
| H2R4R9 | Pan troglodytes | 73 | X |  |
| A0A0D8Q6Z6 | Photobacterium iliopiscarium | 142 |  | X |
| Q6LKN5 | Photobacterium profundum (strain SS9) | 138 |  | X |
| A0A0B2D7R8 | Pseudomonas flexibilis | 160 |  | X |
| A0A1H2G4M6 | Pseudomonas guangdongensis | 159 |  | X |
| A0A1D9IS22 | Pseudomonas sp. BS-2016 | 130 |  | X |
| A0A1C2JS57 | Pseudomonas sp. CO183 | 161 |  | X |
| A0A1E9CHT1 | Pseudomonas sp. HMSC066B11 | 150 |  | X |
| I9BUT4 | Ralstonia sp. PBA | 132 |  | X |
| Q8CIY2 | Rattus norvegicus | 2 | X |  |
| Q9ES45 | Rattus norvegicus | 6 | X |  |
| Q924V1 | Rattus norvegicus | 58 | X |  |
| Q9WV87 | Rattus norvegicus | 63 | X |  |
| Q672K1 | Rattus norvegicus | 66 | X |  |
| Q9ERL1 | Rattus norvegicus | 75 | X |  |
| Q217F0 | Rhodopseudomonas palustris (strain BisB18) | 120 |  | X |
| Q213X6 | Rhodopseudomonas palustris (strain BisB18) | 157 |  | X |
| G2WM87 | Saccharomyces cerevisiae (strain Kyokai no. 7 / NBRC 101557) | 84 | X |  |
| H0GYE0 | Saccharomyces cerevisiae x Saccharomyces kudriavzevii (strain VIN7) | 85 | X |  |
| A0A0L8RHF5 | Saccharomyces eubayanus | 83 | X |  |
| S5I1U4 | Salmonella enterica subsp. enterica serovar Cubana str. CFSAN002050 | 152 |  | X |
| G5R5E0 | Salmonella enterica subsp. enterica serovar Senftenberg str. A4-543 | 144 |  | X |
| S9X736 | Schizosaccharomyces cryophilus (strain OY26 / ATCC MYA-4695 / CBS 11777 / NBRC 106824 / NRRL Y48691) | 88 | X |  |
| S9W1W7 | Schizosaccharomyces cryophilus (strain OY26 / ATCC MYA-4695 / CBS 11777 / NBRC 106824 / NRRL Y48691) | 94 | X |  |
| S9Q5Y9 | Schizosaccharomyces octosporus (strain yFS286) | 89 | X |  |
| S9RMQ1 | Schizosaccharomyces octosporus (strain yFS286) | 91 | X |  |
| S9RC11 | Schizosaccharomyces octosporus (strain yFS286) | 93 | X |  |
| Q04800 | Schizosaccharomyces pombe (strain 972 / ATCC 24843) | 90 | X |  |
| O94727 | Schizosaccharomyces pombe (strain 972 / ATCC 24843) | 92 | X |  |
| D4ZIZ1 | Shewanella violacea (strain JCM 10179 / CIP 106290 / LMG 19151 / DSS12) | 79 |  | X |
| A0A1E2VNV3 | Shigella sp. FC2928 | 145 |  | X |
| A0A0T6YL22 | Sinorhizobium sp. GL2 | 131 |  | X |
| A0A0G2Z753 | Streptococcus agalactiae | 128 |  | X |
| A0A139N579 | Streptococcus cristatus | 127 |  | X |
| A0A081QQ49 | Streptococcus mitis | 102 |  | X |
| Q8CZ28 | Streptococcus pneumoniae (strain ATCC BAA-255 / R6) | 129 |  | X |
| A0A0T8LBW3 | Streptococcus pneumoniae | 162 |  | X |
| Q82R51 | Streptomyces avermitilis (strain ATCC 31267 / DSM 46492 / JCM 5070 / NBRC 14893 / NCIMB 12804 / NRRL 8165 / MA-4680) | 122 |  | X |
| D9XXE4 | Streptomyces griseoflavus Tu4000 | 111 |  | X |
| A0A1J4NQ17 | Streptomyces mangrovisoli | 121 |  | X |
| A7E3M1 | Takifugu rubripes | 10 | X |  |
| A7E3L7 | Takifugu rubripes | 38 | X |  |
| H2T6E0 | Takifugu rubripes | 53 | X |  |
| H2S6G6 | Takifugu rubripes | 60 | X |  |
| Q7T1Q1 | Takifugu rubripes | 70 | X |  |
| K0S9N3 | Thalassiosira oceanica | 96 | X |  |
| K0TEX7 | Thalassiosira oceanica | 97 | X |  |
| B8BT25 | Thalassiosira pseudonana | 81 | X |  |
| B8LDU6 | Thalassiosira pseudonana | 82 | X |  |
| B8CG47 | Thalassiosira pseudonana | 95 | X |  |
| A0A191W8T2 | Vibrio anguillarum | 151 |  | X |
| A0A0H3Q3D0 | Vibrio cholerae B33 | 140 |  | X |
| A0A0C3IDM4 | Vibrio mytili | 141 |  | X |
| A0A090PSW4 | Vibrio ponticus | 133 |  | X |
| U2ZWL6 | Vibrio proteolyticus NBRC 13287 | 136 |  | X |
| F9RQA6 | Vibrio scophthalmi LMG 19158 | 137 |  | X |
| A0A061Q462 | Vibrio sp. JCM 19052 | 135 |  | X |
| A0A1A6IXN5 | Vibrio tasmaniensis | 134 |  | X |
| A0A0H0XYF8 | Vibrio vulnificus CladeA-yb158 | 139 |  | X |
| A0A0P7FDA8 | Xanthomonas sp. Mitacek01 | 117 |  | X |
| A0A0T9TLP1 | Yersinia kristensenii | 143 |  | X |
